# Relevance of standardized phase angle and bioelectrical impedance vectors in hospitalized older patients: A cohort study

**DOI:** 10.1002/ncp.11339

**Published:** 2025-06-17

**Authors:** Nahara Oliveira Lima da Silva Diniz, Jarson P. Costa‐Pereira, Claudia Porto Sabino Pinho Ramiro, Maria da Conceição Chaves de Lemos, Fabiana Cristina Lima da Silva Pastich Gonçalves, Alcides da Silva Diniz, Márcia Marília Gomes Dantas Lopes, Ana Paula Trussardi Fayh, Ilma Kruze Grande de Arruda

**Affiliations:** ^1^ Graduate Program in Nutrition, Department of Nutrition Federal University of Pernambuco Recife Brazil; ^2^ Hospital of Clinics Federal University of Pernambuco Recife Brazil; ^3^ Brazilian Company of Hospital Services, EBSERH Recife Brazil; ^4^ Department of Nutrition Federal University of Health Sciences of Porto Alegre Natal Brazil; ^5^ Postgraduate Program in Applied Sciences to Women's Health, Health Sciences Center Federal University of Rio Grande do Norte Natal Brazil

**Keywords:** aging, bioelectrical impedance vector analysis, body composition, phase angle, survival

## Abstract

**Background:**

The prognostic significance of standardized phase angle (StPhA) and bioelectrical impedance vector analysis (BIVA) remains unclear among hospitalized older individuals. Our study aimed to investigate the prognostic significance of StPhA and BIVA patterns concerning overall survival in hospitalized older patients.

**Methods:**

A prospective cohort study was conducted among older adults hospitalized in medical or surgical wards. Single‐frequency bioelectrical impedance analysis (BIA) was conducted. Using the raw BIA measurements (resistance and reactance), BIVA plots were graphed and StPhA was calculated. In addition, patients underwent assessments for anthropometry, malnutrition, and muscle strength via handgrip strength test. Follow‐up was conducted for up to 24 months after the initial data collection to determine the incidence of mortality as the outcome.

**Results:**

A total of 168 patients were included in this analysis. They were mostly men, with a median age of 68 years. Onco‐hematological diagnoses were the most frequent. Patients with low StPhA exhibited significantly lower body mass index and fat mass (%) (*P* < 0.05). Analysis of BIVA confidence showed that patients with low StPhA and nonsurvivors exhibited a significant downward shift along the y‐axis, indicating less cell mass. BIVA ellipses demonstrated that patients with low StPhA and nonsurvivors were mainly concentrated in the cachexia and anasarca quadrants. Low StPhA was an independent predictor of mortality (hazard ratio_adjusted_ = 2.28; 95% CI, 1.05–4.97).

**Conclusion:**

Our study highlights the prognostic significance of StPhA and demonstrates the clinical relevance of using BIVA to estimate body composition/nutrition phenotypes in hospitalized older patients.

## INTRODUCTION

Aging, especially when combined with chronic diseases, leads to increased fat mass and loss of skeletal muscle, heightening the risk of abnormal body composition and nutrition impairments in hospitalized older adults.^1^, ^2^, ^3^, ^4^, ^5^ In this context, accurate nutrition assessment is crucial, as these changes are associated with adverse outcomes such as frailty, falls, reduced quality of life, and higher mortality.^6^, ^7^, ^8^, ^9^, ^10^

Bioelectrical impedance analysis (BIA) is a doubly indirect method used to estimate body composition at the bedside.^11^, ^12^ BIA is feasible because of its relative cost‐effectiveness, ease of application, interpretation, portability, and suitability as a bedside approach.^11^, ^12^ However, BIA results can be influenced by several factors, including extreme body mass, abnormalities in hydration status, and typically require population‐specific, sex‐specific, and device‐specific equations to estimate body composition.^12^, ^13^ These assumptions pose additional challenges for clinical practice. On the other hand, raw values from BIA, including resistance (R) and reactance (Xc), can be used to compute a cellular membrane integrity index called phase angle (PhA), using the equation^14^, ^15^:

$$\text{arctangent}\frac{\text{Reactance}(\text{Xc})}{\text{Resistance}(R)}\times \frac{180{}^{\circ}}{\pi }$$

PhA reflects cellular integrity and serves as an additional marker of fluid distribution.^14^ Studies demonstrate a moderate‐to‐strong association between PhA and muscle strength, mass, and “quality” while inversely correlating with fat compartments.^14^, ^15^, ^16^, ^17^ These properties position PhA as a valuable prognostic biomarker in clinical practice.^15^ Although PhA is a relevant biomarker, it is also sensitive to abnormal conditions, such as hydration imbalances, underlying clinical conditions, and technical factors related to BIA devices.^18^ As a result, PhA is not typically used for diagnostic purposes. In this context, standardized PhA (StPhA) can be applied to minimize some of these sources of error.

StPhA converts PhA into *z* scores, allowing for more comparable results by adjusting for factors such as age, sex, and potential clinical condition.^12^ In addition, bioelectrical impedance vector analysis (BIVA) was introduced to reduce the errors associated with using predictive regression equations to estimate body composition in BIA.^19^ BIVA provides an impedance components assessment (Xc and R) plotted as vectors, offering a more accurate evaluation of “body composition” without relying on predictive models.^19^ RXc graphs involve plotting vectors using raw BIA parameters, standardized by height (R/H and Xc/H), and have been used in various clinical populations assessing prognostic significance.^20^, ^21^, ^22^, ^23^

Despite these promising approaches, there is still a lack of research exploring StPhA and BIVA in hospitalized older patients. This gap is significant given their increased vulnerability to adverse changes in nutrition status, hydration, and cellular health, potentially affecting health outcomes. Further investigation could potentially support the clinical use of both StPhA and BIVA, reinforcing their value in managing and monitoring this at‐risk population. Therefore, our study aimed to investigate the prognostic significance of StPhA concerning overall survival in hospitalized older patients. Additionally, we explored BIVA patterns descriptively to assess their potential relationship with the same outcome.

## METHODS

### Study design and participants

This is a secondary analysis of previous prospective cohort studies that assessed the nutrition status of older patients hospitalized for multiple causes.^24^, ^25^, ^26^ Inclusion criteria were men and women aged ≥60 years, diagnosed with any clinical condition, and admitted to either surgical or medical wards at the Hospital das Clínicas de Pernambuco, Recife, Brazil. Data collection occurred between May 2021 and August 2023. All data collection was conducted by four trained and skilled dietitians as part of their hospital routine care. Specifically, they assessed functional status using the Barthel Functioning Scale, anthropometry including calf circumference (CC), nutrition status using the Mini Nutritional Assessment Short Form (MNA‐SF), body composition estimated through BIA, and muscle strength via handgrip strength (HGS) measurements to be subsequently detailed. All nutrition and functional measurements were collected only at baseline.

Patients classified as critically severely ill, unable to respond to questionnaires, or unable to undergo nutrition/anthropometric evaluation were excluded from the study. The study was approved by the Institutional Ethics Committee in compliance with the Declaration of Helsinki, and resolution no. 466/2012 of the National Health Council (approval number: 5.558.401). All participants provided written informed consent.

### Clinical and nutrition variables

All measurements occurred up to 72 h after hospital admission. Sociodemographic and clinical data were collected through interviews and verified using electronic medical records. Data on age, sex, comorbidities, clinical diagnoses, number of medications during hospitalization, anthropometry, and functionality were used for analysis purposes. Comorbidities included systemic arterial hypertension, type 2 diabetes mellitus, and chronic kidney disease, all of which were diagnosed by a specialist physician and documented in the patients' electronic medical records. Functional dependence was assessed using the Barthel Functioning Scale. This scale is a measure of a patient's ability to perform basic activities of daily living independently. It assigns scores ranging from 0 to 100, with higher scores indicating better independence. Scores ≤75 suggest a higher degree of dependence, meaning the individual may require assistance with daily tasks such as bathing, dressing, feeding, and mobility.^27^ For anthropometry, body weight (kg) and height (m) were measured to calculate body mass index (BMI; kg/m^2^). CC was measured using an inelastic tape. The measurement was taken at the largest part of the calf, at the point of maximum circumference. Patients were positioned in a standardized manner, seated with both knees and ankles bent at a 90° angle to the floor. Measurements were conducted twice, and the mean value was recorded for analytical purposes. For this study, we only used unadjusted CC. The MNA‐SF was used to evaluate nutrition status. Those scoring ≥12 were considered to have a normal nutrition status, whereas those scoring ≤12 were classified as abnormal.^28^ Mortality incidence (our outcome of interest) was obtained from electronic medical records at the end of the study (up to 24 months after initial data collection). Efforts were made to contact patients or their relatives via telephone to cross‐verify the data.

### BIA, BIVA, StPhA, and muscle strength

A single‐frequency BIA (Biodynamics 310 50 kHz model) was employed to estimate body composition. Patients adhered to a 2‐h fasting period based on the hospital's meal intervals. During the assessment, they lay in a dorsal decubitus position on a bed, free of metallic objects. The test used four electrodes placed on the right wrist and ankle. Appendicular lean soft tissue (ALST) was calculated using the Barbosa‐Silva et al equation^29^ based on the R and Xc data obtained from a single‐frequency BIA. ALST was normalized by height^2^ to obtain the ALST index. This BIA model also provides fat mass. However, its applicability among older patients remains a topic of future investigation, as the specific algorithms and equations are not provided by manufacturers.

BIVA software developed by Piccoli et al^30^ was used to generate confidence and tolerance ellipses based on BIA raw values. Using mean components of the R/H and Xc/H within a specified group,^30^ we determined the 95% CI for vector means. Interpretation of confidence ellipses was conducted by comparing their lengths along the major axis.^31^ Significantly different positions of two bioimpedance mean vectors in the RXc plane (*P* < 0.05) were identified if their 95% confidence ellipses did not overlap.^32^ Tolerance ellipses provided graphical analysis of individual points or three ellipses (median, third quartile, and 95th percentile), encapsulating 50%, 75%, and 95% of individual points, respectively.^30^ The positioning within the BIVA tolerance ellipses allows for body composition/nutrition phenotypes interpretation based on vector migration. The left side of the ellipse is associated with greater cell mass and better hydration status, whereas the right side is indicative of lower cell mass. BIVA plots categorize individuals into four quadrants based on RXc parameters. The upper right quadrant represents dehydrated and/or lean individuals, presenting with high R and Xc. The lower right quadrant was identified in individuals with cachexia, presenting with low Xc and high R. The lower left quadrant indicates overhydration, with low R and Xc, suggesting fluid retention or anasarca. Lastly, the upper left quadrant was identified in muscular individuals, featuring high Xc and low R, indicative of greater muscle mass and better cellular health.^32^ The central region represents individuals with normal hydration and body composition/nutrition phenotypes, enclosed within the 50% tolerance ellipse. Values beyond the 75% tolerance threshold suggest significant deviations from normality. A visual representation of BIVA ellipses will be provided later in this manuscript.

StPhA was also calculated and derived from raw BIA values (R and Xc). It was calculated using the equation

$$\mathrm{StPhA}=\frac{\text{PhA}-\text{mean PhA for reference population}}{\text{SD for reference population}}$$

Values <−1.65 were classified as low StPhA.^33^ This cutoff is considered “universal” for our population because StPhA is transformed into a *z* score, which accounts for variations across different age and sex groups.

Muscle function was determined by muscle strength, using the HGS test measured by a JAMAR digital dynamometer, following pre‐established techniques.^34^ Patients were instructed to grip the handgrip as forcefully as possible for 5 s, using their dominant hand during the expiration phase. Three attempts were conducted, with verbal encouragement and a 1‐min interval between each attempt. The highest value from the three HGS attempts was used for data analysis.^34^ “Muscle quality” (ie, muscle‐specific strength) was measured through the adapted muscle quality index, using the ratio of HGS to ALST.^35^

### Statistical analyses

Normality of continuous data was assessed through Shapiro‐Wilk test. Normal data were described as mean ± SD. Nonnormal data were described as median and interquartile range (IQR). Categorical variables were described as frequency (*n*, %). Comparisons were made using independent Student *t* test, Mann‐Whitney *U* test, Pearson chi‐square test, or Fisher exact test, as appropriate. From univariate categorical analysis, based on the outcome of interest (ie, overall mortality), prevalence ratios (PRs) and relative risk (RR) with corresponding 95% CIs were estimated. PR was used as a measure of association for cross‐sectional analyses, quantifying the proportion of individuals based on their StPhA classification. RR was applied in time‐to‐event analysis (mortality). These measures were chosen to provide a more interpretable initial effect size in our population of interest.

A Kaplan‐Meier curve was constructed to assess the association between StPhA and survival (significant if log‐rank *P* < 0.05). Subsequently, Cox proportional hazards regression was conducted both in crude and adjusted models to assess the independent association between StPhA and mortality during follow‐up. The proportional hazards assumption was tested using Schoenfeld residuals, and no significant violation was observed (StPhA *P* = 0.17). For BIVA, analyses were performed using the BIVA software (2002), by the Hotelling *T* ^2^ test, and univariate analysis (*F* test) to identify their statistical differences between groups.^30^ The mean group vectors of R and Xc normalized for H are graphically displayed in ellipses with tolerance intervals of 50%, 75%, and 95% according to the values of a reference population. Values outside the 75% ellipse are considered abnormal, as they may capture a broader range of abnormal values that may be clinically relevant.^36^, ^37^ For all analyses, statistical significance was set at 5%.

## RESULTS

**Figure 1:**
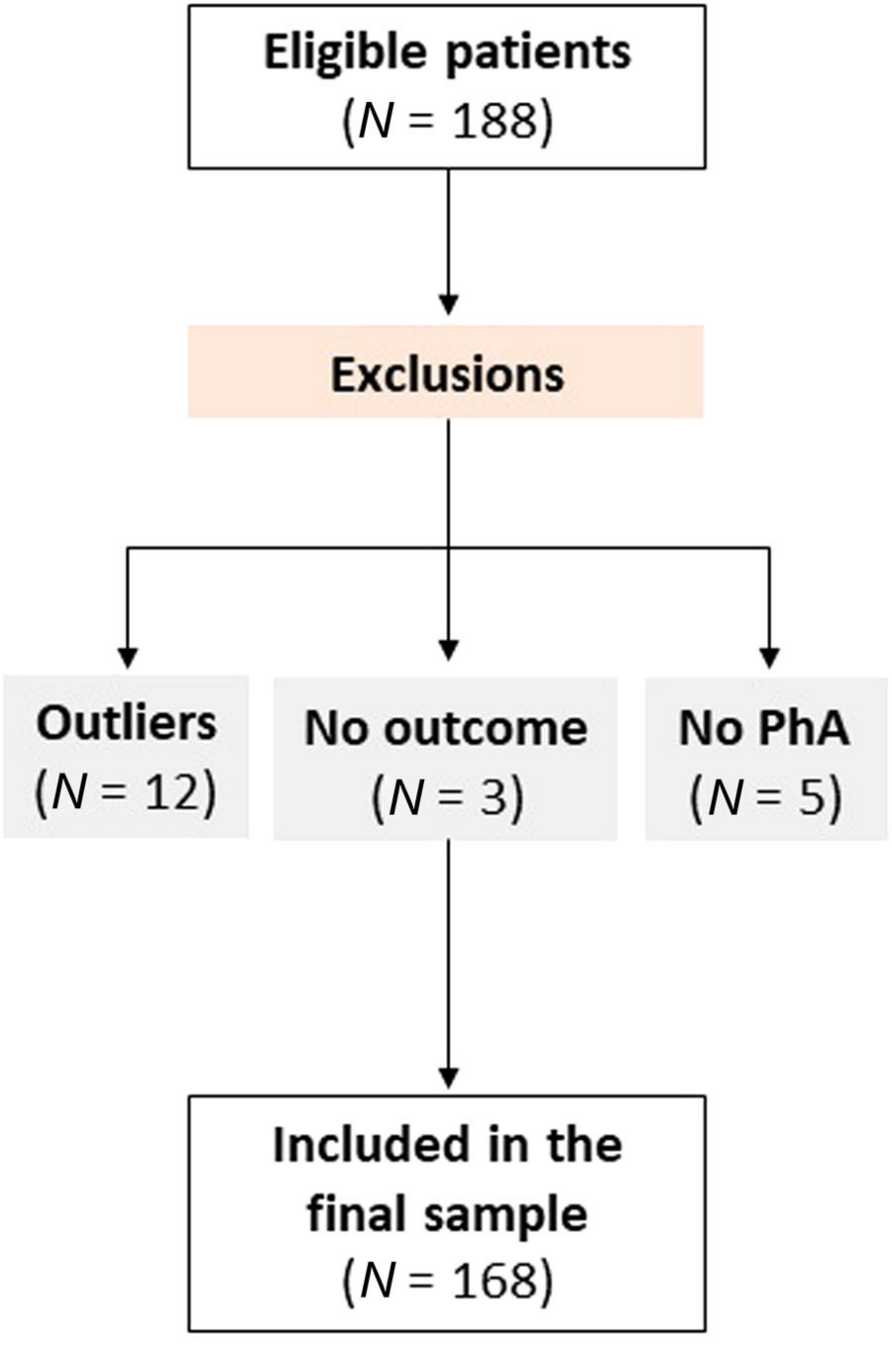
Study flowchart. PhA, phase angle.

Following exclusions (Figure 1), this secondary analysis included 168 older hospitalized patients. The median age was 68 years (IQR = 64–74.8), and 55.4% were male. Onco‐hematological diagnoses were the most frequent (42.9%). When comparing individuals with normal vs low StPhA, no differences were observed for sex distribution, age, clinical diagnoses, comorbidities, polypharmacy, and functional dependence based on Barthel index scores. Patients were followed during their hospitalization and after discharge, over a median period of 17 months (IQR = 14.9–17.9). Patients with low StPhA had significantly higher mortality incidence during follow‐up (14.8% vs 33.3%; *P* = 0.012) (Table 1). Patients with low StPhA had lower values of BMI, fat mass (%), and lower scores of MNA‐SF (all *P* values <0.05). No differences were observed for CC, HGS, muscle mass, and quality (Table 2).

**Table 1:** Characteristics of hospitalized older patients (*N* = 168).

Variables	Overall	Normal StPhA (>−1.65)	Low StPhA (<−1.65)	PR/RR (95% CI)	*P*
*n* (%)	168 (100)	54 (32.1)	114 (67.9)	–	–
Males, *n* (%)	93 (55.4)	25 (46.3)	68 (59.6)	1.19 (0.96–1.48)	0.10^a^
Age, median (IQR), y	68 (64–74.8)	68 (64–75)	68 (64–74)	–	0.78^b^
Diagnoses, *n* (%)					0.47^a^
Genitourinary tract	17 (10.1)	6 (11.1)	11 (9.6)	–	–
Gastrointestinal tract	15 (8.9)	5 (9.3)	10 (8.8)	–	–
Cardiovascular and respiratory	48 (28.6)	18 (33.3)	30 (26.3)	–	–
Onco‐hematological	72 (42.9)	23 (42.6)	49 (43.0)	–	–
IPDs, neuro and rheumatological	16 (9.5)	2 (3.7)	14 (12.3)	–	–
SAH, *n* (%)	122 (72.6)	44 (81.5)	78 (68.4)	0.82 (0.66–1.00)	0.07^a^
2DM, *n* (%)	72 (42.9)	27 (50.0)	45 (39.5)	0.87 (0.70–1.08)	0.19^a^
CKD, *n* (%)	16 (9.5)	5 (9.3)	11 (9.6)	1.02 (0.72–1.44)	0.93^a^
Number of medications, *n* (%)	4 (3;6)	4 (3;6)	4 (3;6)	–	0.37^a^
Functional dependence, *n* (%)	50 (29.8)	13 (24.1)	37 (32.5)	1.13 (0.92–1.40)	0.27^b^
Mortality, *n* (%)^c^	46 (27.4)	8 (14.8)	38 (33.3)	1.33 (1.09–1.61)	0.012^a^

Abbreviations: 2DM, type 2 diabetes mellitus; CKD, chronic kidney disease; IPD, infectious and parasitic disease; IQR, interquartile range; PR, prevalence ratio (for baseline categorical data); RR, relative risk; SAH, systemic arterial hypertension; StPhA, standardized phase angle.

^a^ Pearson chi‐square test (frequencies *n* [%]).

^b^ Mann‐Whitney *U* test (median and IQR).

^c^ RR was the only variable assessed during follow‐up.

**Table 2:** Nutrition characteristics of hospitalized older patients (*N* = 168).

Variables	Normal StPhA (>−1.65), median (IQR)	Low StPhA (<−1.65), median (IQR)	*P* a
BMI, kg/m^2^	27.7 (22.7–30.2)	24.5 (21.3–27.7)	0.042
CC, cm	33 (30–36)	32 (30–35)	0.82
HGS, kg	20 (14–28)	19 (14–27)	0.72
Fat mass, %	35.6 (30.5–40.9)	31.1 (24–37.7)	0.015
ALSTI, kg/m^2^	16.9 (14.3–18.5)	16.9 (14.9–18.6)	0.78
MQI_BIA_	0.30 (0.20–0.41)	0.26 (0.18–0.38)	0.29
MNA‐SF	11 (8–14)	10 (7–12)	0.049

Abbreviations: ALSTI, appendicular lean soft tissue index; BIA, bioelectrical impedance analysis; BMI, body mass index; CC, calf circumference; HGS, handgrip strength; IQR, interquartile range; MNA‐SF, Mini Nutritional Assessment Short Form; MQI, muscle quality index; StPhA, standardized phase angle.

^a^ Mann‐Whitney *U* test (median and IQR).

**Figure 2:**
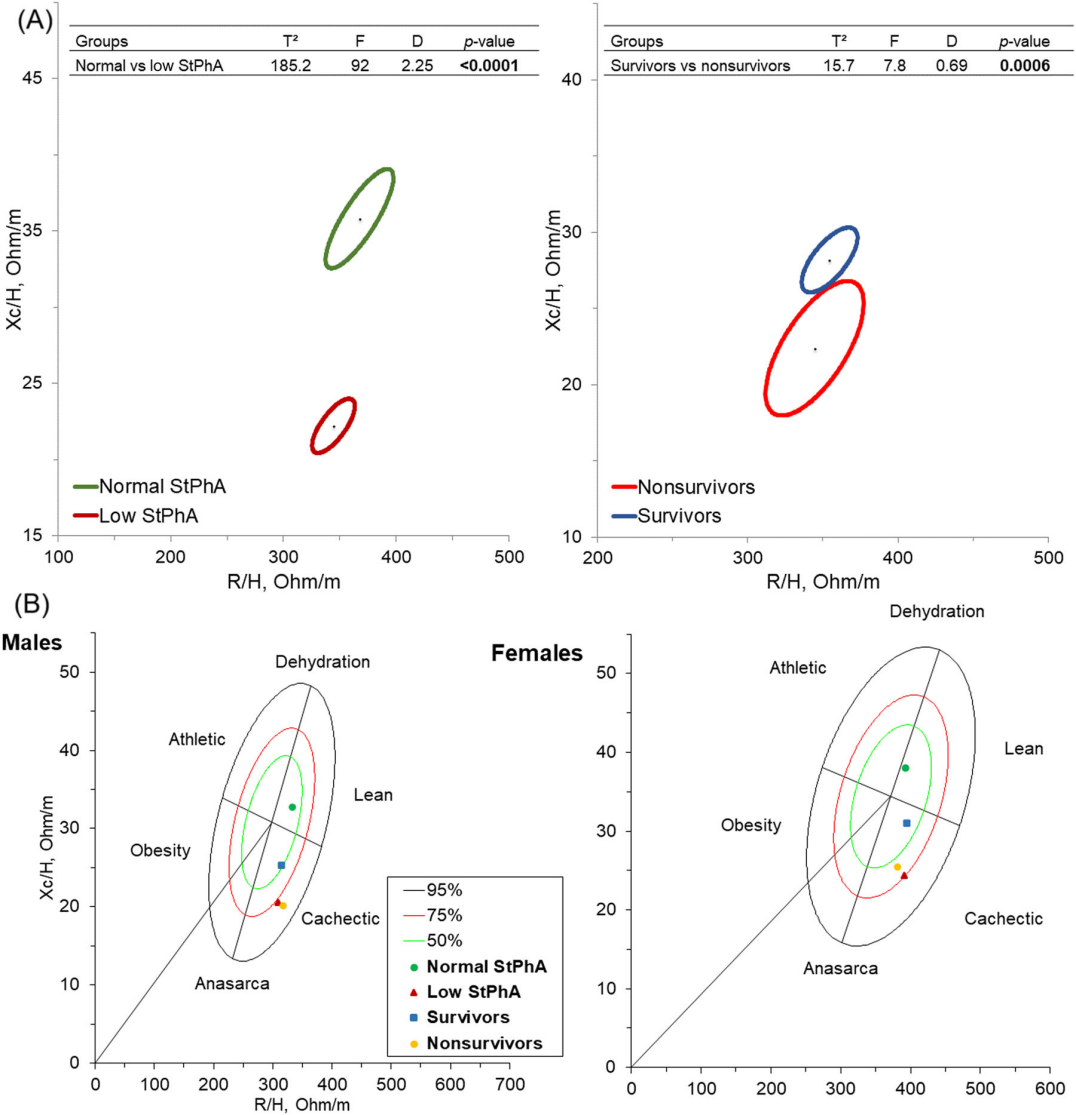
(A) Bioelectrical impedance vector analysis confidence ellipses comparing vectors between patients with normal vs low StPhA and between survivors vs nonsurvivors. (B) Bioelectrical impedance vector analysis tolerance ellipses based on StPhA and survival status, stratified by sex. Mean vector positions for each group are plotted. StPhA, standardized phase angle.

BIVA confidence ellipses demonstrated that patients with low StPhA, as well as nonsurvivors, exhibited lower values and a downward shift along the y‐axis (*P* < 0.001), indicating lower cell mass (Figure 2A). Figure 2B shows the tolerance ellipse BIVA plots. Males with low StPhA were predominantly located in the abnormal cachexia quadrant, whereas those with normal StPhA values were positioned within the lean, normal quadrants. A similar pattern was observed for survival: nonsurvivors were concentrated in the abnormal cachexia region. Among women, those with low StPhA values were positioned near the borderline of the abnormal cachexia quadrant (at 75%). Although survivors were located within ideal quadrants, nonsurviving women tended to cluster near the abnormal cachexia and anasarca region, although still within the “normal” classification range (<75%).

**Figure 3:**
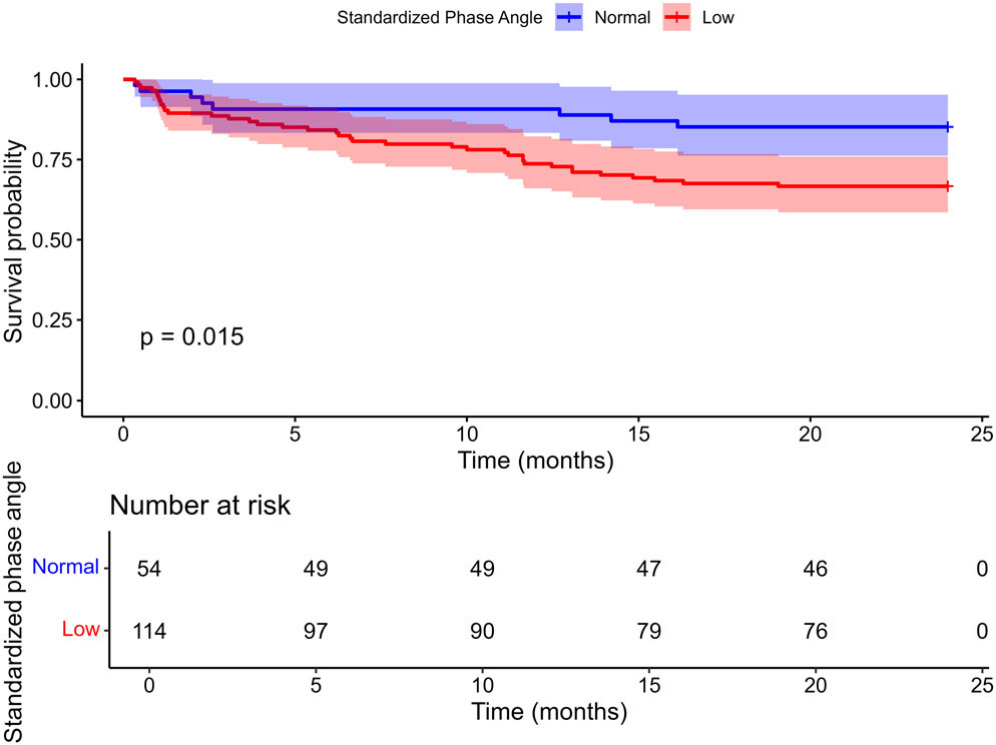
Kaplan‐Meier curve demonstrating survival probabilities across standardized phase angle classifications of hospitalized older patients.

Figure 3 provides a KM curve demonstrating the association between StPhA and overall survival. Patients with low StPhA had significantly poor survival (log‐rank *P* = 0.015). Higher values of StPhA were independently and inversely associated with mortality. For each increase in StPhA, there was a corresponding 31% decrease in the hazard of mortality. Additionally, as a categorical variable, low StPhA was independently associated with a higher hazard of mortality (adjusted hazard ratio = 2.28; 95% CI, 1.05–4.97; Table 3).

**Table 3:** Cox regression analysis: associations between StPhA and overall mortality of hospitalized older patients (*N* = 168).

Variables	HR (95% CI)	*P*	HR_adjusted_ (95% CI)	*P*
Model 1				
StPhA	0.71 (0.61–0.83)	<0.001	0.76 (0.65–0.89)	0.001
Model 2				
Low StPhA	2.48 (1.16–5.32)	0.020	2.33 (1.07–5.07)	0.033

*Note*: Model 1 used a continuous variable, and model 2 used a categorical variable. Models were adjusted for age, clinical diagnoses, number of medications, Barthel scores, body mass index, and Mini Nutritional Assessment scores. StPhA was already sex‐stratified.

Abbreviations: HR, hazard ratio; StPhA, standardized phase angle.

## DISCUSSION

The novelty of our study lies in its focused exploration of the prognostic value of StPhA, complemented by BIVA patterns in a specific population of older hospitalized patients with diverse clinical conditions that reflect real‐world hospital settings. This research enhances our understanding of these methods and highlights their potential for clinical application in assessing patient health and risk. Our main findings demonstrated that low StPhA was an independent predictor of mortality, irrespective of whether it was used as a continuous or categorical variable. Furthermore, we observed that both patients who died and those with low StPhA exhibited significantly downward positions on the BIVA y‐axis confidence plots. Tolerance ellipses confirmed that these patients were predominantly characterized by cachexia (ie, less cell mass) and fluid overload (anasarca).

The prognostic value of StPhA has been explored in various clinical populations, yielding conflicting results.^38^, ^39^, ^40^ For instance, one study identified it as an independent predictor of prolonged hospital stays among critically ill patients,^38^ whereas another study involving patients following acute myocardial infarction demonstrated a limited prognostic significance.^39^ However, in a hospitalized cohort of patients with cancer, low StPhA emerged as a predictor of poor survival,^40^ aligning with our findings. Such investigations across diverse clinical populations are a foundational step in stratifying risk and guiding clinical and nutrition decisions. Prior studies have demonstrated that PhA can be influenced by various factors, including sex, age, and abnormal BMI, particularly extreme adiposity.^41^, ^42^ Within this context, using StPhA may help mitigate the influence of age and sex, thereby enhancing its clinical relevance.

Our additional findings revealed that patients with lower StPhA exhibited significantly lower BMI and fat mass (%) values. Given that StPhA may serve as an indicator of overall cell mass and oxidative stress^14^, ^43^ it is possible that patients with lower fat mass and BMI may present with a poorer clinical status and, consequently, lower StPhA. Additionally, because fat tissues are poor electrical conductors (low Xc), this may also explain the observed relationship with lower StPhA. Our additional findings on MNA‐SF scores demonstrated that patients with low StPhA had significantly lower scores, suggesting a higher risk or existing state of malnutrition. Furthermore, the incidence of mortality was twice as high in patients with low StPhA. In this context, StPhA is reinforced as a promising prognostic marker that can be used in both clinical and research settings to assess the risk of mortality among older hospitalized patients. It also indicates poor nutrition status, characterized by higher fat levels and an increased (risk of) malnutrition.

Our BIVA analysis demonstrated significant differences between groups. Individuals with low StPhA or those who died were mainly categorized as having cachexia or anasarca, albeit with modest differences between sexes. These findings align with our initial results, which indicated that patients with low StPhA exhibited compromised fat mass (%), BMI, and worse malnutrition scores. Previous studies have demonstrated the prognostic impact of cachexia in clinical populations.^44^ However, its operational diagnosis is still complex, potentially leading to differences in methods and limiting its application in clinical practice.^45^ Although it is not yet long‐established whether BIVA can truly serve as a surrogate marker for the clinical cachexia phenotype, if confirmed, it could be more easily implemented in clinical practice. This remains a future goal of our research group.

Despite a promising approach, we acknowledge that our specific device and population currently lack reference data for BIVA patterns, which may skew our findings. To address this, we have adopted previously published reference data available in the BIVA software (ie, traditional reference values).^30^ Establishing reference values specific to our BIA model (Biodynamics 310) using a sample of young and healthy individuals is a future goal of our research group.

The predominance of cachexia in our study can be partially explained by the population's characteristics. We centered on older patients with exacerbated chronic conditions requiring hospitalization, with nearly half of them diagnosed with oncological conditions. In such a vulnerable population, cachexia is likely to occur because of its association with inflammation, characterized by proteolysis, cell autophagy, and lipolysis.^46^ Cachexia represents a particularly severe manifestation, posing significant treatment challenges and sometimes irreversible consequences.^46^ Similarly, the position near the anasarca quadrant can be understood within the context of inflammation. Reactive oxygen species in an inflammatory state compromise cellular integrity, disrupting cellular fluid balance and resulting in extracellular abnormal fluid retention.^14^, ^47^ This underscores the complex interplay between inflammation and fluid regulation in our patient population, highlighting the need for comprehensive management strategies.

Although previous studies have demonstrated the feasibility and relevance of employing BIVA among clinical populations,^19^, ^20^, ^21^, ^22^, ^23^ there remains a scarcity of studies focusing on nutrition phenotypes derived from BIVA in a population of older patients hospitalized for multiple causes. Despite the heterogeneity of this population, we acknowledge that this may reflect the reality of hospital settings. In this regard, we believe that using BIVA in such cohorts may aid in stratifying the risk associated with older age, coupled with chronic and acute conditions that can significantly impact body composition and, consequently, nutrition status.

Our study is not without limitations. The use of a single‐center observational design, convenience sampling, and a small sample size prevents the establishment of causal relationships and the generalization of findings to broader populations. Additionally, for plotting BIVA ellipses, we relied on reference values from populations aged ≤85 years. Given the limited number of patients older than this age, our results may be somewhat biased, potentially affecting accurate assessments. Furthermore, not all patients or family members were successfully reached by telephone to verify the incidence of mortality (only 71.4%). As a result, the reliance on medical records may have led to underreporting, potentially contributing to discrepancies in follow‐up.

Logistical constraints prevented all patients from being followed for the same duration, potentially introducing biases in mortality incidence. Additionally, conducting more stratified time‐to‐event analyses (eg, censoring at 12 months) was not feasible owing to the limited number of events. Although intraclass and interclass analyses were not conducted because of the unavailability of detailed data required for such calculations, all dietitians involved in data collection were highly skilled and experienced in nutrition assessments. They followed standardized protocols as part of routine hospital care, ensuring consistency and quality in the data collected. However, the absence of these analyses represents a limitation and potential source of bias.

We also used a BIA equation validated for our older adult population but not specifically for our BIA device, which may introduce bias and partially explain the lack of differences observed in StPhA concerning ALST index. It is important to note that our BIA model does not yet provide a specific equation, which presents challenges previously raised in this manuscript. Additionally, we relied solely on HGS as a marker of muscle strength, a measure that is highly muscle‐group specific and may not fully capture age‐related strength loss, which tends to be more pronounced in the lower limbs.^48^ This may also partially explain the lack of significant differences between groups. Limitations present opportunities for future research with larger, more representative samples, allowing for a more accurate and robust analysis.

## CONCLUSIONS

This study adds to the growing body of evidence regarding the application of BIVA for assessing abnormal phenotypes related to body composition. Additionally, we emphasize that StPhA among hospitalized older patients is an independent predictor of mortality up to 24 months after hospital discharge. Our study highlights the value of using BIVA in clinical care and demonstrates the prognostic value of a simplified index based on raw BIA data, diminishing the need for reliance on predictive equations.

## AUTHOR CONTRIBUTIONS

Nahara Oliveira Lima da Silva Diniz, Jarson P. Costa‐Pereira, Maria da Conceição Chaves de Lemos, Ilma Kruze Grande de Arruda, and Claudia Porto Sabino Pinho Ramiro contributed to the conception and design of the research; Jarson P. Costa‐Pereira and Nahara Oliveira Lima da Silva Diniz acquired data; Jarson P. Costa‐Pereira, Alcides da Silva Diniz, Márcia Marília Gomes Dantas Lopes, and Ana Paula Trussardi Fayh contributed to data analysis. Jarson P. Costa‐Pereira and Nahara Oliveira Lima da Silva Diniz wrote the manuscript. All the authors critically reviewed, interpreted, and approved the final version of the manuscript.

## CONFLICT OF INTEREST STATEMENT

Ana Paula Trussardi Fayh reports receiving a grant for research from Prodiet Medical Nutrition. Jarson P. Costa‐Pereira has previously received travel costs from Fresenius Kabi as part of the Jumpstart Clinical Nutrition Program. The remaining authors declare no conflict of interests.

## Data Availability

The data that support the findings of this study are available from the corresponding author upon reasonable request.
